# Efficient 2D Neck Model for Simulation of the Whiplash Injury Mechanism

**DOI:** 10.3390/bioengineering11020129

**Published:** 2024-01-29

**Authors:** Diamantino Henriques, Ana P. Martins, Marta S. Carvalho

**Affiliations:** 1 UNIDEMI, Department of Mechanical and Industrial Engineering, NOVA School of Science and Technology, Universidade Nova de Lisboa, 2829-516 Caparica, Portugal; dfm.henriques@campus.fct.unl.pt (D.H.); apc.martins@campus.fct.unl.pt (A.P.M.); 2 Laboratório Associado de Sistemas Inteligentes, LASI, 4800-058 Guimarães, Portugal

**Keywords:** impact biomechanics, whiplash, optimization

## Abstract

Whiplash injuries, mainly located in the neck, are one of the most common injuries resulting from road collisions. These injuries can be particularly challenging to detect, compromising the ability to monitor patients adequately. This work presents the development and validation of a computationally efficient model, called Efficient Neck Model—2D (ENM-2D), capable of simulating the whiplash injury mechanism. ENM-2D is a planar multibody model consisting of several bodies that model the head and neck with the same mass and inertia properties of a male occupant model in the 50th percentile. The damping and non-linear spring parameters of the kinematic joints were identified through a multiobjective optimization process, solved sequentially. The TNO-Human Body Model (TNO-HBM), a validated occupant model for rear impact, was simulated, and its responses were used as a reference for validation purposes. The root mean square (RMS) of the deviations of angular positions of the bodies were used as objective functions, starting from the bottom vertebra to the top, and ending in the head. The sequence was repeated until it converged, ending the optimization process. The identified ENM-2D model could simulate the whiplash injury mechanism kinematics and accurately determine the injury criteria associated with head and neck injuries. It had a relative deviation of 8.3% for the head injury criteria and was 12.5 times faster than the reference model.

## 1. Introduction

Road accidents still cause a significant number of fatalities every year. In 2020, the United States of America (U.S.) recorded over 40,000 deaths despite the implementation of improved security systems in vehicles. However, the number of fatalities decreased significantly with the distance traveled due to the constant improvement in vehicle safety. This has resulted in the lowest number of deaths in the last decade [1].

The field of vehicle passive safety has a long history, dating back to the early days of automobiles. It has been a highly active area of research and development for many decades, with a particular emphasis in the later part of the 20th century and continuing until today [2]. Crashworthiness has been primarily concerned with the study of impact biomechanics ever since DeHaven’s groundbreaking research [3]. DeHaven is an air crash survivor who is often referred to as the father of crashworthiness. He conducted initial studies on injury biomechanics and published the first work on this topic. Stapp [4], on the other hand, developed an experimental program to evaluate human tolerance to extreme accelerations to establish limits.

To overcome the ethical and moral issues raised throughout history using human volunteers, animals, and human cadavers, Anthropomorphic Test Devices (ATDs), usually called dummies, were developed to be more than human mechanical surrogates to be used in the evaluation of occupant protection in a crash event. The first ATD was developed by Sierra Engineering in 1949 for ejection seat testing by the U.S. Air Force, until which bags of flour were used to simulate the occupant response [5].

ATDs are designed to be biofidelic to mimic human physical characteristics of size, shape, mass, stiffness, energy absorption, and dissipation so that their mechanical responses during vehicle collision conditions correspond to human dynamic responses. They are instrumented to measure accelerations and loading that a vehicle occupant withstands during a crash event. The evaluation of the protection that seating systems offer to passengers is performed through the biomechanical dynamic responses measured in ATDs, which can be representative of the amount of injury risk. To be a reliable device, the ATD must satisfy several requirements like biofidelity, repeatability, reproducibility, durability, and calibration standards [5]. For a review of developments in the area of impact biomechanics, the interested reader is referred to [6].

The National Highway Traffic Safety Administration (NHTSA) is a U.S. federal agency created in 1970 that focuses on transportation safety. The agency is responsible for writing and enforcing regulations that license road vehicle manufacturers. The first frontal crash tests performed by NHTSA took place in 1978, which contributed to the improvement of automobiles sold in the USA. Later, in 1996, the European New Car Assessment Program (EuroNCAP) was created to evaluate the new road vehicle models according to the protocols created by EuroNCAP, with the tests being carried out voluntarily by the manufacturers [7].

As cars have become more advanced, their safety features have improved significantly. Despite this, the number of injuries resulting from rear-end collisions continues to be a major concern in the United States. In fact, in 2020 alone, there were over 417,062 injuries attributed to rear-end collisions [1]. This type of collision causes a sudden backward rotation of the head, called whiplash, which results in injuries located along the neck and in soft tissues such as intervertebral discs, ligaments, and muscles [8,9]. Symptoms of these injuries typically include pain in the neck, back, and shoulders. Some less common complaints are numbness in the upper limbs, dizziness, blurred vision, tiredness, depression, and anxiety. While most patients can recover quickly, around 40% still experience symptoms after 3 months, and between 2 and 4.5% suffer from permanent injuries [8]. Therefore, while these injuries are not typically fatal, they result in a loss of quality of life and high medical monitoring costs [9].

**Figure 1:**
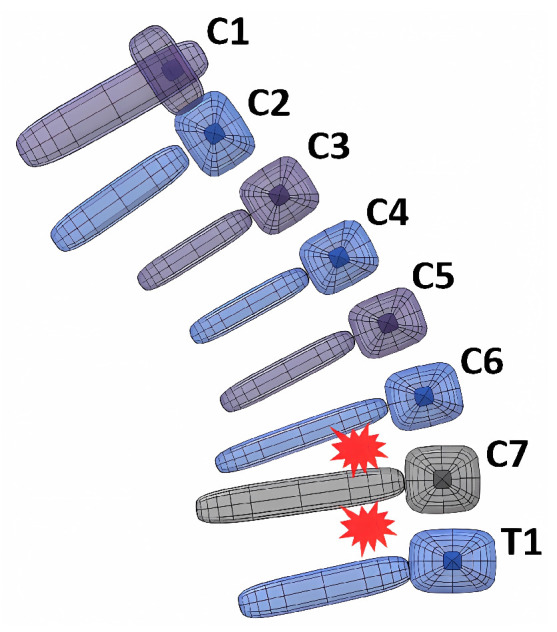
Localization of injuries during retraction phase.

There is a wide variety of injuries located in the vertebrae, which are particularly difficult to detect on X-rays, traditional, and magnetic resonance imaging, which in many cases compromise the adequate medical monitoring of patients [8]. Through an experimental test carried out by Panjabi et al. [10], it was discovered that whiplash injuries occur mainly during the retraction phase in the inter-vertebral spaces between T1 and C6, as presented in Figure 1. Injuries occur due to these vertebrae having greater extension during retraction, although the maximum extension occurs in the head.

Multibody dynamics provides a methodological framework for the representation of complex structural arrangements in crashworthiness, as used in the development of vehicle models for frontal and side impacts [11]. It also provides accurate and efficient methodologies for the description of the large rigid body motion of the anatomical segments and mechanisms of the ATD [12,13]. However, non-linear finite element methods have an unsurpassed ability to represent all details of the structural deformations being directly linked to geometric modeling software that enables the generation of complex models with simplicity [14,15,16]. Multibody models are more efficient and less computationally expensive than finite element models for handling large movement dynamics of ATD models. Therefore, they are preferred over ATD finite element models in such scenarios. When it comes to developing criteria for neck injuries, measuring the stress and strain that muscles and tissue ligaments can withstand is crucial [17,18,19,20]. Advanced neck finite element modeling can provide dynamic responses of the system that are not possible with equivalent multibody models. For analysis that requires long run time, Schwartz et al. [21] developed a simplified model in finite element to become an additional computational tool focused on the study of injury biomechanics.

The objective of this work was the development and validation of a computationally efficient neck model for the rear-end collision scenario. Since this collision occurs in the sagittal plane, it was modeled in the framework of multibody dynamics, a planar model with the same mass and inertia properties of the 50th percentile male occupant model called TNO-Human Body Model (TNO-HBM), which was developed and validated for a varied set of impact situations, including rear-end collisions [22,23]. The fact that the developed model is computationally more efficient than any 3D ATD numerical model [24] makes it advantageous in its use in optimization processes where it is usually necessary to carry out a high number of simulations [11]. The use of the efficient model combined with optimization methodologies for the identification of its design variables allows for its use in extensive parametric analysis to improve the design of the seat mechanisms that mitigate whiplash injuries.

## 2. Numerical Modeling of the ENM-2D

### 2.1. Geometry and Inertia

**Figure 2:**
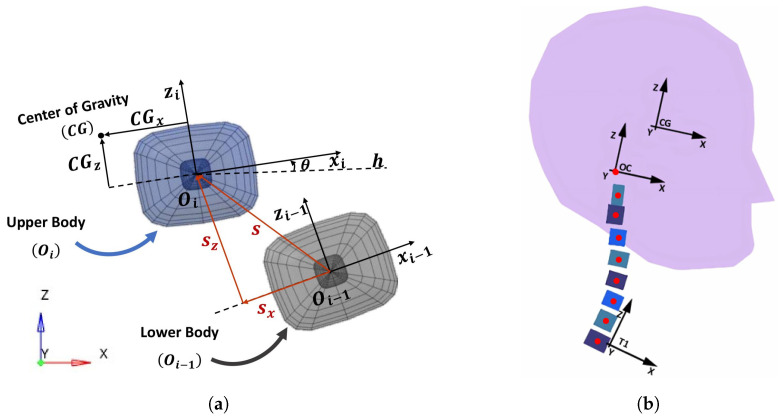
ENM- 2D model and reference frames used in its construction. (**a**) Bodies’ reference frames. The upper body position (**S**) was defined in the lower body local reference frame (x_*i*−1_ and z_*i*−1_). The upper body CG was defined in its local reference frame (x_*i*_ and z_*i*_) and the horizontal (**h**) was defined in the global reference frame (XYZ). (**b**) Complete model.

The geometry of the ENM-2D was developed by reproducing the head and cervical vertebrae bodies, which are part of a 50th percentile male ATD for rear-end collisions. Starting from the bottom in T1, the position of the upper body (body *i*) is defined in relation to the lower body (body i−1 ), as presented in Figure 2a. For that, the center point (O_*i*−1_) of body i−1 serves as the origin of the axis Z_*i*−1_ and X_*i*−1_, and the center point ( $O_{i}$ ) of body *i* is located at (S_*x*_; S_*z*_) of body i−1 . The orientation of the body *i* is defined by θ , measured relative to the horizontal (h). For simplification, consecutive bodies are connected with revolute joints. These revolute joints should ideally be located at the instantaneous axis of rotation (IAR) observed during the rear-end collisions. However, the IAR location changes during the motion of the vertebrae [25], so to keep the simplicity of the model, it was decided to locate the IAR of the upper bodies on the O_*i*−1_ point, as represented by the red marks depicted in Figure 2b. The figure illustrates the origin of the reference frame for T1, which is initially positioned at (4, 19) mm from the global reference frame, as well as the occipital (OC) reference frame and the head center of mass (CG) reference frame.

The properties of mass and positioning of all the bodies are summarized in Table 1.

### 2.2. Stiffness and Damping

The revolute joints that connect consecutive bodies were modeled with a set of torsional springs and dampers resembling the ENM-2D. For the constitutive functions of these springs and dampers, it was necessary to use the values found in [26,27] for an initial estimate of the parameters.

The study conducted by Camacho et al. [26] provided the moment–rotation curves for spring stiffness. These curves were then applied to vertebrae to determine their moment–rotation curves. The resulting curves were given by Equation (1), where *M* represents the applied moment (Nm) and θ represents the rotation between the bodies (rad):

(1) $$M=A\left(e^{\theta B}-1\right)$$

The coefficients *A* (Nm) and *B* (−) are dependent on the connection and of the spring rotation sense. The values used for the coefficients are summarized in Table 2.

The damping properties were based on those used in an occupant model developed in 2008 [27], for which were assumed equal spring–damper parameters for all body connections. The damping coefficient was defined as a function of the time, equal to 1.8 Nms/rad during the initial 50 ms, and then it increased to simulate the muscular reaction of the occupant. For the ENM-2D, a constant damping value *C* of 1.8 Nms/rad was used since the active muscular reaction was not modeled in the TNO-HBM version used for the simulation reference scenario described hereafter.

### 2.3. Reference Configuration and Simulation Results

**Figure 3:**
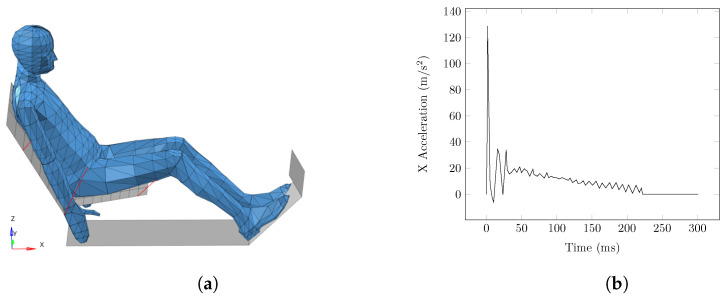
Setup used for the simulation of the LAB test with the TNO-HBM. (**a**) Position of the TNO-HBM (reference model). (**b**) Acceleration pulse applied on the platform.

To validate the ENM-2D model, the reference responses were defined by simulating the LAB sled test scenario in MADYMO, using the TNO-HBM as the occupant model. The LAB sled test consisted of using post-mortem subjects belted to a rigid seat subjected to a forward acceleration pulse [23]. Therefore, the TNO-HBM was placed on a rigid seat defined by two rigid planes, the seat-back and the base, which were oriented at 25° and 10°, respectively, with the horizontal plane, as depicted in Figure 3a. The model was kept attached to the seat by seat belts on the thorax, hip, and thigh, similar to the experimental LAB test, limiting any deformation to the neck and head. Then, the acceleration pulse shown in Figure 3b was imposed on the seat, which mimicked a rear-end collision at 10 km/h [23]. The results of the dynamic response measurements of head and neck bodies were calculated in MADYMO and used as references to validate the ENM-2D.

**Figure 4:**
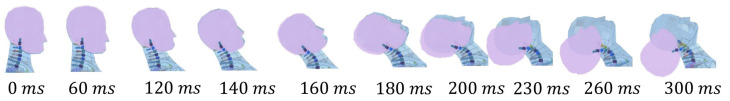
Kinematics: ENM-2D (initial configuration) vs. TNO-HBM (reference). Both models’ frames overlap, and ENM-2D is displayed in the front.

To reproduce this scenario with the ENM-2D model, the reference T1 displacement was applied to the ENM-2D T1 vertebra. Selected frames of overlapped kinematics for both models illustrate the mechanism of whiplash injury, as depicted in Figure 4.

In the beginning, the kinematics of the ENM-2D model were very similar to the reference results, but after 180 ms, the differences between the results became clear. At the end time of the simulation, the highest difference between the models was verified, around 70° for the rotation of the head, and for the rotation of the head relative to T1. Therefore, an identification process was developed for the ENM-2D to reduce differences between the models. This process tunes selected stiffness and damping parameters through an optimization procedure.

## 3. Identification Process

The objective functions were the root mean square (RMS) of the difference between the angular position of each ENM-2D’s body and its respective reference, which are the results from the simulation of the TNO-HBM. This involved selecting 40 design variables that correspond to the stiffness and damping parameters of each revolute joint to minimize the RMS.

**Figure 5:**
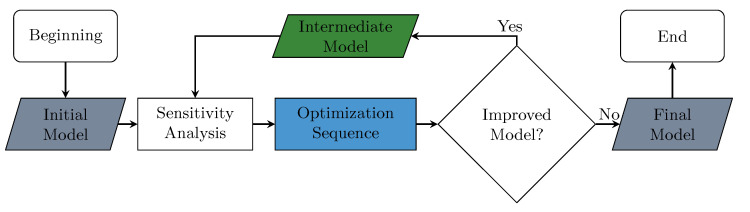
Process used for the identification of optimal models.

Since this is a multiobjective problem, first, a sensitivity analysis was performed using the ENM-2D initial model (named V1). In the analysis, each variable design was perturbed by 25%, and its impact on the RMS of the angle values was evaluated. It was observed during the sensitivity analysis that the variables associated with a specific revolute joint had a significant impact on the rotation of the bodies over that joint, while the bodies below it were not affected as much. As a result, the approach to solve this multiobjective optimization problem involved the sequential resolution of single-objective optimization problems. The flowchart outlining this approach can be seen in Figure 5. The sequence began at the bottom vertebra. The model minimized the RMS of each vertebra from C7 to C0. The optimization method used in this process was the Modified Method of Feasible Directions (MMFDs) [28]. However, other gradient-based algorithms could also be used for optimization, such as the ones mentioned in [29,30]. Alternatively, one could use genetic or hybrid algorithms to bring about changes in the optimization process, as suggested in [30].

In each step of the design process, the variables that had the most influence on the model’s response were selected. These variables are listed in Table 3. To prevent excessive variation that could affect other results, their range was limited to 25%. However, it was expected that the optimal values would be close to the initial model, as these parameters were based on experimental values.

After solving the first sequential optimization problem, the identified model was renamed (V2), and the sequential optimization process was repeated as many times as necessary until converging to the final model, as depicted in the flowchart in Figure 5. The process finished when the improvements were not considerable compared with the previous model results; in this case, it was necessary to solve four sequences until the final model (V5) was obtained.

**Figure 6:**
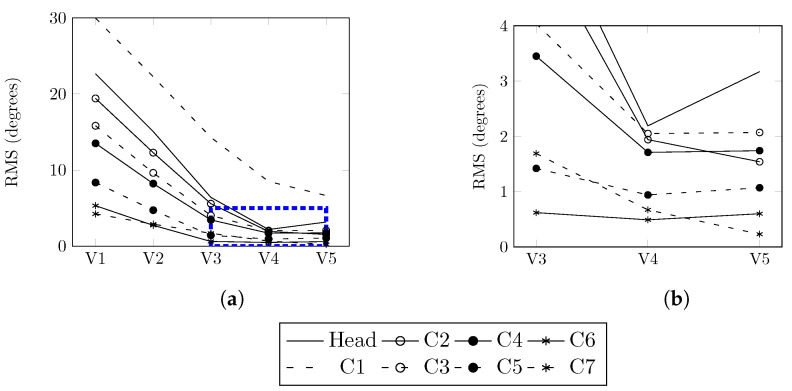
Evolution of RMS values during the optimization sequence. (**a**) RMS values for models V1 to V5. (**b**) Detailed RMS values for models V3 to V5.

Figure 6 shows a decrease in all RMS values for the differences relative to angles after the first three sequences. During the fourth sequence, there was a notable increase in the angle of the head and a slight increase in the angles of the C4, C5, and C6 vertebrae. However, improvements were seen in the remaining vertebrae. As a result of these observations, the identification process was concluded.

**Figure 7:**
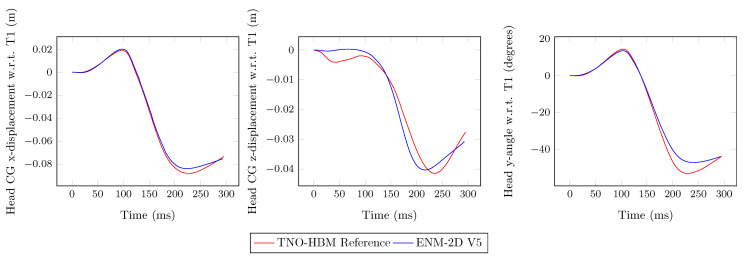
Head CG linear displacement components and angular displacement with respect to T1: ENM-2D vs. reference.

In Figure 7, the results of the head kinematics are plotted and compared to the ENM-2D V5 model with its reference (TNO-HBM). The *y*-rotation, i.e., flexion/extension, of the head relative to T1 was similar to the reference up to 150 ms. The *x*-displacement, i.e., anterior/posterior, of the head CG relative to T1 corresponded satisfactorily with the reference. However, for the *z*-displacement, i.e., superior/inferior, the same did not occur, and throughout the simulation, a significant difference emerged.

**Figure 8:**
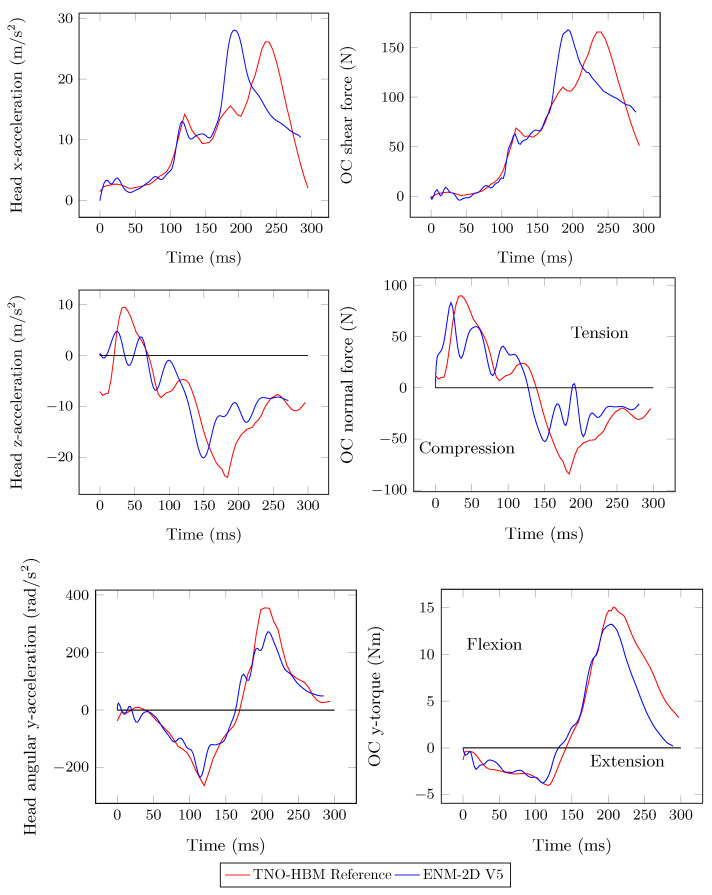
Head acceleration and resulting forces in the OC: ENM-2D V5 vs. TNO-HBM (reference).

Considerable improvements were achieved for angular acceleration and torque responses from the identified model ENM-2D V5, which are now closer to the TNO-HBM reference responses, as shown in Figure 8. The peak of *x*-acceleration occurred slightly earlier than the reference, but was still close. The same is true for the shear force. However, in the case of *z*-acceleration and the normal force, the initial peak value was not replicated. Instead, several local peaks of lower intensity were observed.

## 4. Modification of the Model and Application of the Optimization Process

To improve the model’s performance, we prioritized enhancing the displacement of the head CG. However, optimizing the *z*-offset would likely result in a worse outcome for the *x*-direction. This is because the head’s center of gravity depends on both T1 displacement (which was imposed) and vertebrae rotation due to the interconnected revolute joints in the body.

**Figure 9:**
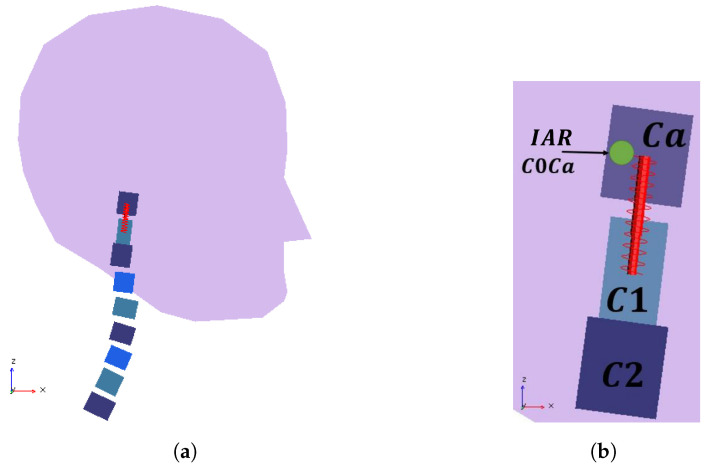
ENM-2D modified with the implementation of an additional mass-spring–damper set. (**a**) Complete modified model. (**b**) Detail of the additional linear spring–damper.

To achieve independence between the head’s *x*- and *z*-displacements, the model required some modifications. The procedure consisted of introducing a spring–damper assembly between the head and C1 vertebra, as presented in Figure 9. This spring–damper set was also used to model the translation between consecutive vertebrae.

To include the set, a massless auxiliary body called Ca was added at (19.3, 142) mm. Ca and C1 have the same orientation. They are connected by a translation joint that only allows displacement in the direction connecting points *O* of C1 and Ca. The revolute joint between C0/C1 was relocated to C0/Ca while maintaining its initial position. To maintain model simplicity, a linear spring elasticity ( $k_{v}$ ) and constant damping coefficient ( $c_{v}$ ) were used. After simulating the trial, 10 kN/m and 100 Ns/m were chosen as initial values for $k_{v}$ and $c_{v}$ , respectively. $k_{v}$ and $c_{v}$ were then added to the previous 40 design variables for the new identification process, which follows the flowchart in Figure 5. Instead of minimizing the RMS values of the rotation of the eight bodies, the objectives were to improve the occipital’s *x*- and *z*-displacements (RMS OC) and the rotation of the head (RMS C0). Table 4 lists the design variables and objective functions for each step in the optimization sequence.

**Figure 10:**
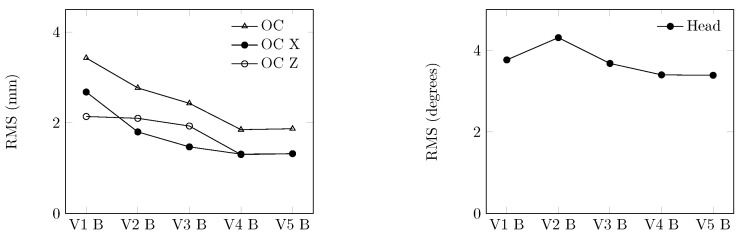
Evolution of RMS values used in the optimization sequence of the modified model.

The plots depicted in Figure 10 illustrate the evolution of the RMS OC and the RMS C0. As the RMS values had already converged, only one optimization sequence was carried out. The model that was identified, ENM-2D V5 B, included an additional linear spring and damper with final values of 1620 N/m for $k_{v}$ , and 100 Ns/m for $c_{v}$ . The remaining design parameters for the model that has been identified can be located in Table 5.

**Figure 11:**
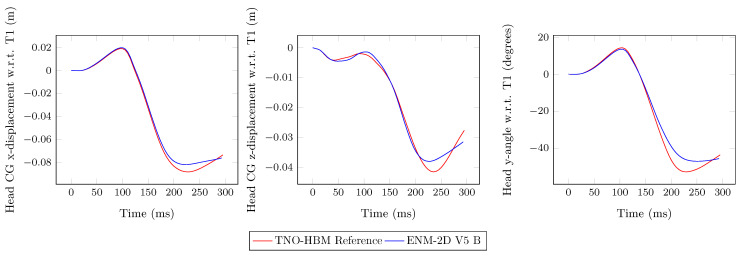
Head CG linear displacement components and angular displacement with respect to T1: reference vs. final ENM-2D.

**Figure 12:**
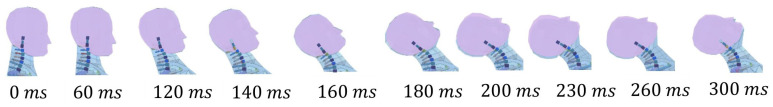
Kinematics: ENM-2D (final) and TNO-HBM (reference). Both models’ frames overlap, and ENM-2D is displayed in the front.

In Figure 11, we observe a slight improvement for the head *y*-rotation and a better correspondence for the *z*-displacement of the head, although it comes at a cost of a slight worsening of the *x*-offset. The overlapping kinematic frames of the modified ENM-2D with the TNO-HBM (reference) are shown in Figure 12. One can notice that there is a good correspondence between the two models. There was a reduced difference between the displacement of the head and the rotation of the body in both models, which was expected. This correspondence is particularly relevant up to 140 ms, as during this period, there is typically contact between the head and the backrest.

**Figure 13:**
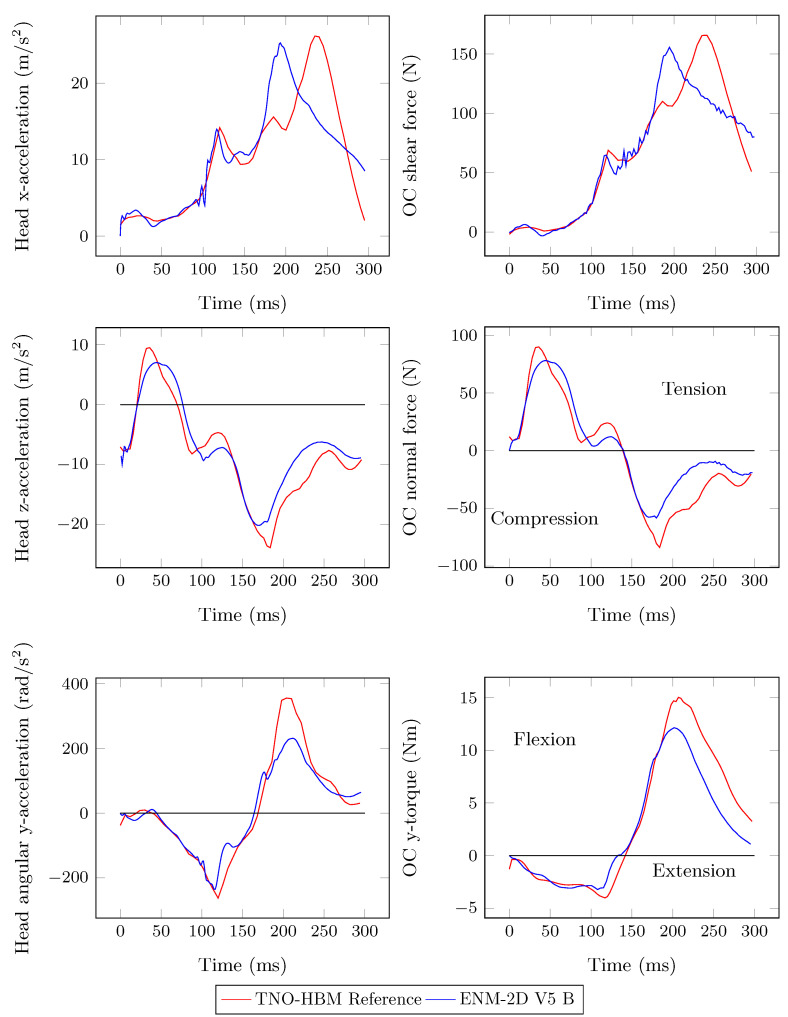
Head acceleration and resulting forces in the OC: ENM-2D V5 B vs. TNO-HBM (reference).

Figure 13 shows the plots related to the forces and acceleration in the head’s CG. The *z*-acceleration and normal force in the head have better correspondences, which were the responses that motivated the modification of the model. The remaining dynamic responses experienced slight changes, mainly in their peak value. After improving the kinematics of the ENM-2D, it is now necessary to access relevant values for calculating the injury criteria.

## 5. Injury Criteria Assessment

The Head Injury Criterion (HIC) [31] is expressed by Equation (2). In this equation, $t_{1}$ and $t_{2}$ represent the initial and final instants during which the acceleration is measured, respectively, and a(t) represents the resultant of the measured acceleration at the head CG. To obtain the highest possible HIC value at the integration time, the time window between $t_{1}$ and $t_{2}$ is selected. For collisions involving direct contact with the head, the contact interval is usually 15 ms, while for collisions that do not involve direct contact with the head (like in the present simulation), the contact interval is 36 ms.

(2) $$HIC=max\left\{\left(t_{2}-t_{1}\right){\left[\frac{1}{t_{2}-t_{1}}\int_{t_{1}}^{t_{2}}a\left(t\right)dt\right]}^{2.5}\right\}$$

The $N_{km}$ criterion, explained through Equation (3), was suggested by Schmitt et al. [32]. It is based on the hypothesis that a neck protection criterion for rear-end collisions should consider a linear combination of normalized shear forces $F_{x}\left(t\right)$ and bending moments $M_{y}\left(t\right)$ at the occipital condyles. The normalization is performed using $F_{int}$ and $M_{int}$ , respectively.

(3) $$N_{km}=\frac{F_{x}\left(t\right)}{F_{int}}+\frac{M_{y}\left(t\right)}{M_{int}}$$

There are four possible load cases of the $N_{km}$ criterion, which are named N_*fa*_, N_*ep*_ , N_*fp*_, and N_*ea*_. The first index indicates whether it is under flexion or extension, which is denoted by *f* or *e*. The second index indicates the direction of the shear force, which is either anterior or posterior, denoted by *a* or *p*. The $F_{int}$ is either −845 N or 845 N, accordingly, if the shear force is posterior or anterior, and the $M_{int}$ is −47.5 Nm or 88.1 Nm if the neck is in extension or flexion, respectively.

The calculations performed here have yielded the HIC_36_ and $N_{km}$ criteria results, which are displayed in Table 6.

The ENM-2D results are similar to those of TNO-HBM in terms of the HIC, with only an 8.3% deviation. However, the ENM-2D model is slightly more conservative, predicting slightly more severe head injuries. In terms of the neck criteria, all of them indicate a good match, except for N_*ep*_. Nevertheless, since both models have very low N_*ep*_ values, the difference is not significant enough to lead to different conclusions regarding neck injuries.

## 6. Discussion

This study aimed to develop and validate an efficient neck model for rear-end collisions. The ENM-2D was developed using a planar multibody dynamics framework, based on geometrical information of the sagittal plane of the 50th percentile male occupant, the TNO-HBM [22,23]. The stiffness of the revolute joints was estimated using moment–rotation curves obtained from experimental research conducted by Camacho et al. [26]. A translational spring–damper set was added to the model to improve *z*-acceleration and normal force in the head CG.

Through an optimization methodology, the parameters of the ENM-2D were identified, using the TNO-HBM dynamic responses to the LAB sled test simulation as a reference. It should be noted that the TNO-HBM had already been validated for the LAB sled test (12 g, Δ V = 10 km/h, rigid seat, no head restraint), whereby the details of this research validation were reported by Van der Horst [23]. The objective functions selected were the RMS of the deviation of the rotation angles between the vertebrae, making this a multiobjective optimization problem. This optimization scheme considered a sequential approach of previous studies from Carvalho et al. [33,34]. The approach aimed to solve a single-objective optimization problem for each pair of vertebrae, rather than using the weighted sum of the objective functions [33], or determining a Pareto Front as conducted successfully by [34]. This sequential approach was used to avoid the computational cost of dealing with 42 design variables. In each step of the optimization problem, only the design variables that impacted the objective function were considered, resulting in less than eight variables per step.

From the observation of the overlapping frames depicted in Figure 12, no differences were noted, as expected, since the deviations in the angular position of the bodies were minimized. The quantitative correspondence between ENM-2D and its reference, as shown in Figure 11 and Figure 13, is particularly relevant up to 140 ms. These results match the findings from Panjabi et al. [10] for the neck S-shape curvature.

Regarding injury criteria, the ENM-2D model is slightly more conservative than the TNO-HBM model when it comes to predicting Head Injury Criterion (HIC), with 8.3% more severe head injury predictions. The neck criteria match well, except for Nep. However, both models have low Nep values, and are thus insignificant to neck injuries, as supported by Schmidt et al. [6].

The ENM-2D is significantly more efficient than the TNO-HBM. When simulating 300 ms of the rear-end impact, the ENM-2D is 12.5 times faster than the TNO-HBM 3D model, even while using the same processor. The ENM-2D’s efficiency makes it ideal for optimization processes requiring a high number of simulations. Therefore, this model enables more efficient computational analysis in comparison to using 3D finite element models, such as for the seat design position and angle of the headrest conducted by Wang et al. [35].

## 7. Limitations

The ENM-2D is a numerical model of the head and neck of a 50th percentile male occupant, considered validated only for the LAB test crash pulse (12 g, Δ V = 10 km/h). However, with the presented methodology, it is entirely possible to model and identify occupant models that represent different genders and percentiles for a given crash scenario. The ENM-2D is a planar model of the sagittal section of the occupant, wherein the most relevant dynamics of the rear-end impact occurs. We should keep in mind that the ENM-2D is a simplified model that should exclusively be used in the preliminary design stages. It is not intended to replace more in-depth numerical modeling and experimental testing in the final design stages.

## 8. Conclusions

In this work, a computational model called ENM-2D was presented. This model can accurately replicate the TNO-HBM response when subjected to the simulation of a sled test, referred to as the LAB test, which was used as a reference. To solve a multiobjective problem involving 40 design variables, the proposed identification methodology involves solving single-objective optimization problems sequentially. The model was modified by introducing a translational spring–damper set, which added two more design variables to the multiobjective optimization problem. With the proposed methodology, the problem was identified successfully. The identified model was found to be capable of reproducing the positioning and head rotation, as well as reasonably determining the injury criteria associated with head and neck injuries. Therefore, the ENM-2D was validated for the rear impact scenario and LAB test crash pulse using the proposed methodology.

In future developments, the ENM-2D model can be integrated with design solutions such as head restraints, airbags, and car interior trims to mitigate injury risks based on the occupant’s head–neck response in rear-end impacts.

## Figures and Tables

**Table 1:** Mass properties and initial position of the bodies.

Body	Mass (kg)	Moment of Inertia (kg m^2^)			Position ^1^ $O_{i}$ rel. to $O_{i-1}$ (mm)		Position ^2^ CG (mm)		Angle θ with the Horizontal (°)
		**Ixx**	**Iyy**	**Izz**	**Sx**	**Sz**	**Gx**	**Gz**	
Head (C0)	4.69	0.018	0.024	0.017	−2	21	27	0.043	−13
C1	0.22	0.001	0.001	0.002	2	16	−7.7	0	−7
C2	0.25	0.001	0.001	0.002	−2	19	−7.7	0	−7
C3	0.24	0.001	0.001	0.002	−1	18	−7.8	0	−7
C4	0.23	0.001	0.001	0.002	2	18	−7.9	0	−12
C5	0.23	0.001	0.001	0.002	3	17	−8.1	0	−17
C6	0.24	0.001	0.001	0.002	6	17	−8.3	0	−23
C7	0.22	0.001	0.001	0.002	7	17	−8.2	0	−26
T1	0.86	0.010	0.010	0.020	0	0	0	0	−26

^1^ T1 is the first body; therefore, the position of each upper body’s local reference frame $\left(O_{i}\right)$ was defined relative to the lower body reference frame $\left(O_{i-1}\right)$ . The coordinates of the position vector (**S**) were defined in the local reference frame of the lower body. ^2^ The coordinates of the CG position are defined in the local body reference frame.

**Table 2:** Stiffness coefficients ^1^ from the experimental research of Camacho et al. [26].

		C0/C1	C1/C2	C2/C3	C3/C4	C4/C5	C5/C6	C6/C7	C7/T1
Extension	$A_{1}$ (Nm)	−0.0095	−0.0095	−0.0037	−0.0068	−0.0027	−0.0126	−0.0125	−0.3105
	$B_{1}$ (−)	−0.3937	−0.3937	−1.0137	−1.1416	−1.6410	−0.9581	−1.2366	−0.6489
Flexion	$A_{2}$ (Nm)	0.0135	0.0135	0.1029	0.0218	0.1130	0.0618	0.1406	0.6084
	$B_{2}$ (−)	0.3052	0.3052	0.4714	0.7503	0.3929	0.5587	0.5607	0.3949

^1^ During extension, coefficients A and B are subscripted as 1, and as 2 during flexion.

**Table 3:** Summary of the design variables (spring coefficients A and B, and damping coefficient C) used in each step of the sequences of the multiobjective problem resolution.

Model ^1^	Objective Functions ^2^ of the Optimization Sequence							
	**RMS C7**	**RMS C6**	**RMS C5**	**RMS C4**	**RMS C3**	**RMS C2**	**RMS C1**	**RMS C0**
V1 (Initial model)	A2_C7/T1 B2_C7/T1 C_C7/T1	A1_C6/C7 B1_C6/C7 C_C6/C7	A2_C5/C6 B2_C5/C6 C_C5/C6	A2_C4/C5 B2_C4/C5 C_C4/C5	A2_C3/C4 B2_C3/C4 C_C3/C4	A2_C2/C3 B2_C2/C3 C_C2/C3	A2_C1/C2 B2_C1/C2 C_C1/C2	A1_C0/C1 B1_C0/C1 B2_C0/C1 C_C0/C1
V2	A2_C7/T1 B2_C7/T1 C_C7/T1	A2_C6/C7 B2_C6/C7 C_C6/C7	A2_C5/C6 B2_C5/C6 C_C5/C6	A2_C4/C5 B2_C4/C5 C_C4/C5	A2_C3/C4 B2_C3/C4 C_C3/C4	A2_C2/C3 B2_C2/C3 C_C2/C3	A2_C1/C2 B2_C1/C2 C_C1/C2	B1_C0/C1 A2_C0/C1 B2_C0/C1 C_C0/C1
V3	A2_C7/T1 B2_C7/T1 C_C7/T1	A2_C6/C7 B2_C6/C7 C_C6/C7	A1_C5/C6 A2_C5/C6 B2_C5/C6 C_C5/C6	A2_C4/C5 B2_C4/C5 C_C4/C5	A2_C3/C4 B2_C3/C4	A2_C2/C3 B2_C2/C3 C_C2/C3	A2_C1/C2 B2_C1/C2 C_C1/C2	A2_C0/C1 B2_C0/C1 C_C0/C1
V4	A2_C7/T1 B2_C7/T1	A2_C6/C7 B2_C6/C7 C_C6/C7	A2_C5/C6 B2_C5/C6 C_C5/C6	A2_C4/C5 B2_C4/C5	A2_C3/C4 B2_C3/C4 C_C3/C4	A2_C2/C3 B2_C2/C3 C_C2/C3	A2_C1/C2 B2_C1/C2 C_C1/C2	A2_C0/C1 B2_C0/C1 C_C0/C1

^1^ Each model V_*i*_ (Intermediate Model in Figure 5) corresponds to the model obtained after one sequence of optimization resolution. ^2^ The objective functions in each step were the RMS of the rotation of each vertebra C_*i*_.

**Table 4:** Summary of the design variables and objective functions used in each step of the sequence of the multiobjective problem resolution for the modified model.

Model	V1 B	V2 B	V3 B	V4 B
**Objective Functions**	**RMS OC**	**RMS OC**	**RMS OC**	**RMS C0**
Design Variables	$k_{v}$ B2_C7/T1 B2_C6/C7 B2_C5/C6 B2_C4/C5 B2_C3/C4	$k_{v}$ B2_C7/T1 B2_C6/C7 B2_C5/C6 B2_C4/C5 B2_C3/C4 B2_C2/C3 B2_C1/C2	$k_{v}$	A2_C0/C1 B2_C0/C1

**Table 5:** Final value of stiffness and damping coefficients obtained for ENM-2D.

Variables	Joints							
	**C0/C1**	**C1/C2**	**C2/C3**	**C3/C4**	**C4/C5**	**C5/C6**	**C6/C7**	**C7/T1**
$A_{1}$ (Nm)	−0.0071	−0.0095	−0.0037	−0.0068	−0.0027	−0.01225	−0.0125	−0.3105
$B_{1}$ (−)	−0.2215	−0.3937	−1.014	−1.142	−1.641	−0.9581	−1.237	−0.6489
$A_{2}$ (Nm)	0.0159	0.0330	0.2512	0.0433	0.2335	0.1037	0.2057	1.322
$B_{2}$ (−)	0.4131	0.7029	0.8907	1.173	0.7685	1.027	0.8521	1.240
*C* (Nms/rad)	2.954	4.395	1.582	1.758	3.516	4.395	3.279	3.516

**Table 6:** Injury criteria in head and neck: TNO-HBM (reference) vs. ENM-2D (final).

Interval	Models	Injury Criteria				
		Head	Neck			
		**HIC_36_**	**N*_ep_***	**N_*fp*_**	**N_*ea*_**	**N_*fa*_**
[0; 300] ms	TNO-HBM	127.1	0.0291	0	0.1596	0.3284
	ENM-2D	137.7	0.0509	0	0.1403	0.3159

## Data Availability

The data presented in this study are available on request from the corresponding author.

## References

[1] Administration N.H.T.S. (2022). Traffic Safety Facts 2020: A Compilation of Motor Vehicle Crash Data. https://crashstats.nhtsa.dot.gov/.

[2] Ambrósio J. (2005). Crash analysis and dynamical behaviour of light road and rail vehicles. Veh. Syst. Dyn..

[3] DeHaven H. (1942). Mechanical analysis of survival in falls from heights of fifty to one hundred and fifty feet. War Med..

[4] Stapp J. (1951). Human exposure to linear acceleration. Aero. Med. Lab. Air Force Rep..

[5] Mertz H.J., Irwin A.L. (2015). Anthropomorphic Test Devices and Injury Risk Assessments. Accidental Injury: Biomechanics and Prevention.

[6] Schmitt K.U., Niederer P.F., Cronin D.S., Morrison B., Muser M.H., Walz F. (2019). Trauma Biomechanics: An Introduction to Injury Biomechanics.

[7] Van Ratingen M., Williams A., Anders L., Seeck A., Castaing P., Kolke R., Adriaenssens G., and Miller A. (2016). The European New Car Assessment Programme: A historical review. Chin. J. Traumatol..

[8] Tameem A., Kapur S., Mutagi H. (2014). Whiplash injury. Contin. Educ. Anaesth. Crit. Care Pain.

[9] Bogduk N. (2011). On cervical zygapophysial joint pain after whiplash. Spine.

[10] Panjabi M., Jonathan G., Jacek C., Kimio N., Jiri D. (1997). Whiplash produces an S-Shaped curvature of the neck with hyperextension at lower levels. Spine.

[11] Carvalho M., Ambrosio J. (2011). Development of generic road vehicle multibody models for crash analysis using an optimisation approach. Int. J. Crashworthiness.

[12] Ambrosio J., Carvalho M., Milho J., Escalante S., Martin R. (2022). A validated railway vehicle interior layout with multibody dummies and finite element seats models for crash analysis. Multibody Syst. Dyn..

[13] Roupa I., da Silva M.R., Marques F., Gonçalves S.B., Flores P., da Silva M.T. (2022). On the modeling of biomechanical systems for human movement analysis: A narrative review. Arch. Comput. Methods Eng..

[14] Kan C.D., Marzougui D., Bahouth G.T., Bedewi N.E. (2001). Crashworthiness evaluation using integrated vehicle and occupant finite element models. Int. J. Crashworthiness.

[15] Carvalho M., Martins A., Milho J. (2018). Validation of a railway inline seating model for occupants injury biomechanics. Int. J. Crashworthiness.

[16] Wu W., Han Z., Hu B., Du C., Xing Z., Zhang C., Gao J., Shan B., Chen C. (2021). A graphical guide for constructing a finite element model of the cervical spine with digital orthopedic software. Ann. Transl. Med..

[17] Meyer F., Humm J., Yoganandan N., Leszczynski A., Bourdet N., Deck C., Willinger R. (2021). Development of a detailed human neck finite element model and injury risk curves under lateral impact. J. Mech. Behav. Biomed. Mater..

[18] Johnson D., Koya B., Gayzik F.S. (2020). Comparison of Neck Injury Criteria Values Across Human Body Models of Varying Complexity. Front. Bioeng. Biotechnol..

[19] Putra I., Iraeus J., Sato F., Svensson M.Y., Thomson R. (2022). Finite element human body models with active reflexive muscles suitable for sex based whiplash injury prediction. Front. Bioeng. Biotechnol..

[20] Yang S., Qu L., Yuan L., Niu J., Song D., Yang H., Zou J. (2022). Finite element analysis of spinal cord stress in a single segment cervical spondylotic myelopathy. Front. Surg..

[21] Schwartz D., Guleyupoglu B., Koya B., Stitzel J.D., Gayzik F.S. (2015). Development of a computationally efficient full human body finite element model. Traffic Inj. Prev..

[22] Happee R., Hoofman M., Kroonenberg A.J.V.D., Morsink P. (1998). A Mathematical Human Body Model for Frontal and Rearward Seated Automotive Impact Loading. J. Passeng. Cars.

[23] Van der Horst M. (2002). Human Head Neck Response in Frontal, Lateral and Rear end Impact Loading: Modelling and Validation. Ph.D. Thesis.

[24] Bortenschlager K., Kramberger D., Barnsteiner K., Hartlieb M., Ferdinand L., Leyer H., Muser M., Schmitt K.U. (2003). Comparison Tests of BioRID II and RID 2 with Regard to Repeatability, Reproducibility and Sensitivity for Assessment of Car Seat Protection Potential in Rear-End Impacts. Stapp Car Crash J..

[25] Panjabi M., Cholewicki J., Nibu K., Babat L., Dvorak J. (1998). Simulation of whiplash trauma using whole cervical spine speciments. Spine.

[26] Camacho D.L., Nightingale R.W., Robinette J.J., Vanguri S.K., Coates D.J., Myers B.S. (1997). Experimental Flexibility Measurements for the Development of a Computational Head-Neck Model Validated for Near-Vertex Head Impact. J. Passeng. Cars.

[27] Himmetoglu S. (2008). Car Seat Design and Human-Body Modelling for Rear Impact Whiplash Mitigation. Ph.D. Thesis.

[28] Kostreva M.M., Chen X. (2000). A superlinearly convergent method of feasible directions. Appl. Math. Comput..

[29] Arora J.S. (2017). Introduction to Optimum Design.

[30] Carvalho M., Ambrósio J., Eberhard P. (2011). Identification of validated multibody vehicle models for crash analysis using a hybrid optimization procedure. Struct. Multidiscip. Optim..

[31] Yoganandan N., Nahum A.M., Melvin J.W. (2014). Accidental Injury: Biomechanics and Prevention.

[32] Schmitt K.U., Muser M.H., Niederer P. A New Neck Injury Criterion Candidate for Rear-End Collisions Taking into Account Shear Forces and Bending Moments. Proceedings of the ESV Conference.

[33] Carvalho M., Milho J., Ambrosio J., Ramos N. (2017). Railway occupant passive safety improvement by optimal design. Int. J. Crashworthiness.

[34] Carvalho M., Martins A.P., Milho J. (2020). Railway seat design for injury mitigation in crash scenario. Int. J. Rail Transp..

[35] Wang Y., Jiang H., Teo E.C., Gu Y. (2023). Finite Element Analysis of Head&ndash;Neck Kinematics in Rear-End Impact Conditions with Headrest. Bioengineering.

